# Evaluation of Procalcitonin, C-Reactive Protein, and Interleukin-6 as Early Markers for Diagnosis of Neonatal Sepsis

**DOI:** 10.1155/2020/8889086

**Published:** 2020-10-01

**Authors:** Emad A. Morad, Rehab A. Rabie, Mohamed A. Almalky, Manar G. Gebriel

**Affiliations:** ^1^Medical Microbiology and Immunology Department, Faculty of Medicine, Zagazig University, Zagazig, Egypt; ^2^Pediatrics Department, Faculty of Medicine, Zagazig University, Zagazig, Egypt

## Abstract

**Background:**

Neonatal sepsis diagnosis is a challenge because of its nonspecific presentation together with low sensitivity of the time-consuming bacterial cultures. So, many sepsis markers, like C-reactive protein (CRP), procalcitonin (PCT), and interleukin-6 (IL-6), are emerging to improve its diagnosis.

**Aim:**

This study was done to investigate the role of CRP, PCT, and IL-6 in promoting the early diagnosis of neonatal sepsis in an attempt to decrease morbidity and mortality.

**Methods:**

This cross-sectional study was conducted on 50 neonates suspected with sepsis enrolled from the neonatal intensive care unit (NICU) of Zagazig University Hospitals, Egypt. Blood cultures for these neonates were done before starting antibiotics. Also, bacterial DNA was revealed from the blood by broad-range 16S rDNA polymerase chain reaction (PCR). Measurements of CRP using the immunoturbidimetry method, PCT using fluorescence immunoassay quantitative method, and IL-6 using commercially available ELISA kit were done to all enrolled neonates.

**Results:**

Forty-one neonates with proved sepsis were found to be positive in blood culture and/or PCR for bacterial 16S rDNA. The most common isolated organisms were *Klebsiella* (61.3%), followed by *E. coli* (9.7%) and *CONS* (9.7%). We detected much significant higher levels of PCT, CRP, and IL-6 in the proved sepsis group than the suspected neonatal sepsis cases (*p* ≤ 0.001, 0.001, and 0.004, respectively). Serum PCT levels showed the highest sensitivity, specificity, PPV, NPV, and accuracy of 97.6%, 89%, 97%, 88.9%, and 96% than other studied sepsis markers.

**Conclusion:**

PCT has satisfactory characteristics as a good marker than IL-6 and CRP for the diagnosis of neonatal sepsis.

## 1. Introduction

Neonatal sepsis is a generalized bacterial infection of the neonate's blood during the first month of life. It is either early-onset (<7 days of birth) or late-onset (>7 days) [1] and it is considered one of the important leading factors for neonatal mortality and morbidity, particularly in developing countries. Consequently, early diagnosis is very important as it helps in beginning antibiotic therapy early, reducing neonatal mortality [2].

The use of conventional blood culture for the isolation of microorganisms is still the gold standard method for diagnosis. However, blood culture can detect the pathogen in only 25% of cases, so it has low sensitivity. It gives false-negative results, especially due to the use of antibiotics. Also, it may give false-positive results due to contamination. In addition, these cultures have a time delay for 2-3 days, and it is difficult to get serial blood samples from neonates [2, 3].

In addition to blood culture, polymerase chain reaction (PCR) amplification of highly conserved DNA sequences found in all bacteria would permit rapid and sensitive detection of bacteria in blood specimens. But its results do not depend on the bacterial viability and so it may still be positive even after antibiotic treatment [4].

Suspected neonates are subjected to broad-spectrum antibiotic treatment empirically until sepsis can be excluded. This antibiotic overuse favors the development of resistance [5]. So, improving the accuracy of the diagnostic tests may decrease the indiscriminative use of antibiotics in cases without sepsis [6]. Recent several studies have included many sepsis markers, like C-reactive protein (CRP), procalcitonin (PCT), and interleukin-6 (IL-6) to improve sepsis diagnosis [3, 6].

C-reactive protein (CRP) is an acute-phase protein synthesized in the liver in response to infection or inflammation. It may be more useful in excluding infections in suspected cases and enable earlier discontinuation of antibiotics. This may be cost-effective, decreasing the development of resistance and hospital stay duration [7].

Procalcitonin (PCT) is the precursor of calcitonin hormone produced in a very low concentration by the C cells of the thyroid gland under normal conditions. It is preferentially induced as an acute-phase reactant in bacterial sepsis. It is produced by macrophages and monocytes of various organs during severe bacterial infection in response to bacterial lipopolysaccharide (LPS), which is a potent inducer of PCT into the circulation [3].

Also, IL-6 is considered one of the most important cytokines, which increase early during the inflammatory cascades. It is produced by monocytes and macrophages in response to bacterial infection. It is required for the survival of the plasma cells, which secrete antibodies and enhance cytotoxic T-cells differentiation. IL-6 is also a hepatocyte stimulating factor that gives rise to the acute-phase response, including CRP [8].

Although numerous papers raised the same issue, this manuscript is one of the few publications about this issue in our locality. Moreover, neonatal sepsis is a challenging problem indeed, and physicians are always in need of methods of prediction and early diagnosis of sepsis to initiate therapy as rapidly as possible to decrease the negative impact on the patient's health and therefore decrease the duration of hospital stay and costs. Therefore, we have carried out our study to search for better markers than CRP for the diagnosis of sepsis.

## 2. Materials and Methods

A cross-sectional study was conducted over six months (January 2019–June 2019) in Medical Microbiology and Immunology Department, Faculty of Medicine, Zagazig University, and the Pediatrics Department, Faculty of Medicine, Zagazig University Hospitals. In this study, we followed the STROBE Statement checklist of items for the observational (cross-sectional) studies.

### 2.1. Patients

The study included 50 neonates with suspected sepsis admitted to the neonatal intensive care unit (NICU), Pediatrics Department, Zagazig University Hospitals.

Any neonate (up to age 28 days) with signs or symptoms of suspected sepsis at the time of admission or who developed sepsis in the hospital during the study period was enrolled in this study. The suggestive clinical manifestation included respiratory distress, apnea, pallor, poor feeding, hypotension, shock, instability of the temperature, lethargy, irritability, and increased oxygen requirement, besides abnormal laboratory findings as abnormal leukocyte count, increased I/T (immature to total neutrophil) ratio, and decreased platelet count. Any neonate with apparent major congenital anomalies, Apgar score less than seven, or on antibiotics therapy before the start of the study was excluded.

All enrolled neonates were subjected to detailed history taken, clinical examination, and some routine laboratory investigations as complete blood count (CBC), blood glucose, serum electrolytes, and arterial blood gases.

This study was approved by the institutional review board (IRB), Faculty of Medicine, Zagazig University. Written informed consent was obtained from all parents of these neonates before enrolling in the study. We followed the ethical principles of the Declaration of Helsinki during the preparation of this study.

### 2.2. Sample Collection

Blood samples were collected under complete aseptic precautions from each study participant before starting general systemic antibiotic treatment. One ml was inoculated immediately into the blood culture bottle; only one blood culture bottle was routinely drawn from each patient. Two ml were collected in plain tubes to separate serum, stored immediately in 3 plastic tubes at −20°C for further CRP, PCT, and IL-6 measurement. Also, one ml was collected in EDTA tubes for PCR.

### 2.3. Blood Culture

About one ml of peripheral venous blood was drawn under complete aseptic precautions from each neonate and inoculated immediately in the neonatal blood culture bottle (Egyptian diagnostic medium, 8 ml volume). Blood culture bottles were incubated at 37°C, for 10 days, then subcultures were done every 48 hours on blood agar and MacConkey agar, which were aerobically incubated at 37°C for 24 hours. The isolated bacteria were identified using standard microbiological techniques [9].

### 2.4. Direct Bacterial DNA Detection in Blood by PCR

DNA was extracted from the blood by QIAamp® DNA Mini kit (Qiagen GmbH, Hilden, Germany) according to manufacture instructions, then subjected to broad-range bacterial 16S rDNA PCR. The primers used were supplied from ThermoFisher Scientific, USA, and their sequences are listed in Table 1. These primers react with highly conserved regions of the bacterial 16S rRNA gene to provide PCR products of about 500 base pairs. The PCR reaction was performed in a total reaction mixture of 50 *μ*l, containing 25 *μ*l of Taq PCR Master Mix (Qiagen GmbH, Hilden, Germany), 2 *μ*l of each primer, 11 of *μ*l RNase free water, and 10 *μ*l of template DNA [5].

The PCR was performed using thermal cycler as follows: initial lysis of cells for 5 min at 94°C, followed by 35 cycles including denaturation for 20 s at 94°C, annealing for 20 s at 58°C, and extension for 60 s at 72°C with a final extension at 72°C for 10 min. The amplified DNA products were separated by electrophoresis on 1.5% agarose gels stained with ethidium bromide and visualized under UV transillumination. The results were compared with negative control (water) and positive control (*E. coli*) in each run.

### 2.5. CRP Measurement

CRP analysis was done using the immunoturbidimetry method. Serum CRP level was measured by the CRP latex agglutination test (Biosystems CRP kit, Catalog No. 31921, Spain) according to the manufacture's guidelines. Serum CRP causes agglutination of the latex particles coated with antihuman CRP. This agglutination was measured by turbidimetry, which is proportional to the CRP concentration.

### 2.6. PCT Measurement

The serum PCT level was measured by the Finecare™ PCT Rapid Quantitative Test (Catalog No. W210). This test is a fluorescence immunoassay used along with the Finecare™ FIA system (Model No. FS-112/FS-113/FS-205) for the quantitative determination of PCT.

The Finecare™ PCT Rapid Quantitative Test is based on fluorescence immunoassay technology, using a Sandwich immunodetection method. When the sample is added into the sample well of the cartridge, the fluorescence-labeled detector PCT antibodies on the sample pad bind to PCT antigens in blood specimens and form immune complexes. As the complexes migrate on the nitrocellulose matrix of the test strip by capillary action, the complexes of detector antibodies and PCT are captured to PCT antibodies that were immobilized on the test strip. Thus, the more PCT antigens in a blood specimen, the more complexes accumulated on the test strip. The signal intensity of fluorescence of detector antibodies reflects the amount of captured PCT.

Each Finecare™ PCT Rapid Quantitative Test cartridge contains internal control that satisfies routine quality control requirements. This internal control is performed each time a patient sample is tested. This control indicates that the test cartridge was inserted and read properly by the Finecare™ FIA system. An invalid result from the internal control causes an error message indicating that the test should be repeated.

### 2.7. IL-6 Measurement

The serum level of IL-6 was measured using Human Interleukin-6 (IL-6) ELISA Kit (Catalog No. 201-12-0091). This ELISA kit is based on the principle of the double-antibody Sandwich technique to detect human IL-6.

### 2.8. Statistical Analysis

The collected data were statistically analyzed using SPSS software (Statistical Package for the Social Sciences software version 25). Quantitative data were represented as the mean value ± standard deviation (SD), median, and range.

The Mann–Whitney test (nonparametric) was used to compare quantitative data. The receiver operating characteristic (ROC) curve was plotted. Sensitivity, specificity, positive predictive value (PPV), negative predictive value (NPV), and accuracy were calculated. Results were considered statistically significant when *p* (probability) values were equal to or less than 0.05.

## 3. Results

This study enrolled 50 neonates with clinically suspected sepsis; 33 males and 17 females, their age ranged 1–16 days with a mean age of 6 ± 2.4, 54% were of low birth weight, 18% had a history of premature rupture of membranes (PROM), and 14% were mechanically ventilated. The mean total leukocytic count (TLC) was 22 ± 8.7 thousand/mm^3^ and the mean Immature/T cell (I/T ratio) was 0.41 ± 0.17 (Table 2).

**Figure 1:**
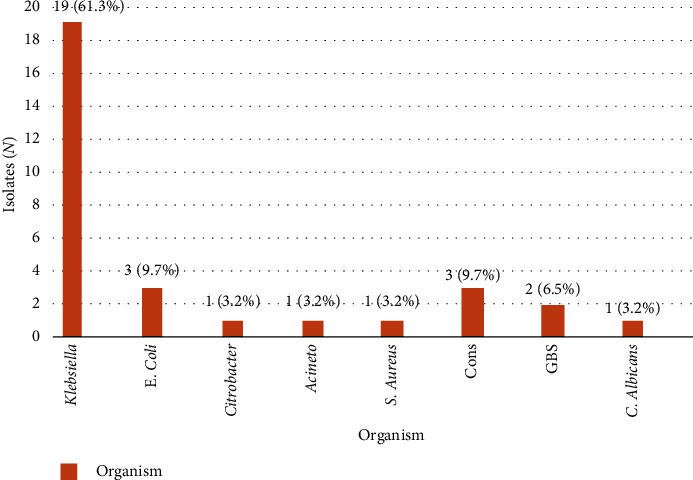
Isolated organisms in infants with neonatal sepsis.

Blood culture was positive in 31 cases; two of them were negative for bacterial 16S rDNA PCR. Gram-negative bacilli were the most common isolated organisms; *Klebsiella* (61.3%) followed by *E. coli* (9.7%), while *Citrobacter* and *Acinetobacter* were less common with 3.2% for each. The most common Gram-positive organisms were *Coagulase-Negative Staphylococci* (CONS) with 9.7%, followed by Group B *Streptococci* (GBS) with 6.5%, then *Staphylococcus aureus* with 3.2%, and finally *Candida albicans* with 3.2% (Figure 1).

Bacterial 16S rDNA was detected in 39 neonates; 10 of them yielded no organisms in blood culture, as shown in Table 3.

The studied neonates were divided into two groups: group 1 included neonates whose samples yielded in blood culture and/or PCR (proved sepsis *n* = 41), and group 2 included neonates suspected of having sepsis with negative both blood culture and PCR results (suspected sepsis *n* = 9).

When the results of blood culture were compared with total proved cases, the blood cultures showed sensitivity 75.6%, specificity 100%, PPV 100%, NPV 47.4%, and accuracy 80% (Table 4). When the results of PCR with proved sepsis were compared, PCR showed sensitivity 95.1%, specificity 100%, PPV 100%, NPV 81.8%, and accuracy 96% (Table 5).

Analyzing the results of sepsis markers revealed that the median procalcitonin (PCT) level of all the studied neonates was 10.4 ng/ml and the range was (0.1–50.6) ng/ml, median CRP serum level was 48 mg/dl and ranged 4–140 mg/dl and IL-6 ranged from 5–120.8 pg/ml with median serum level 36.1 pg/ml.

**Figure 2:**
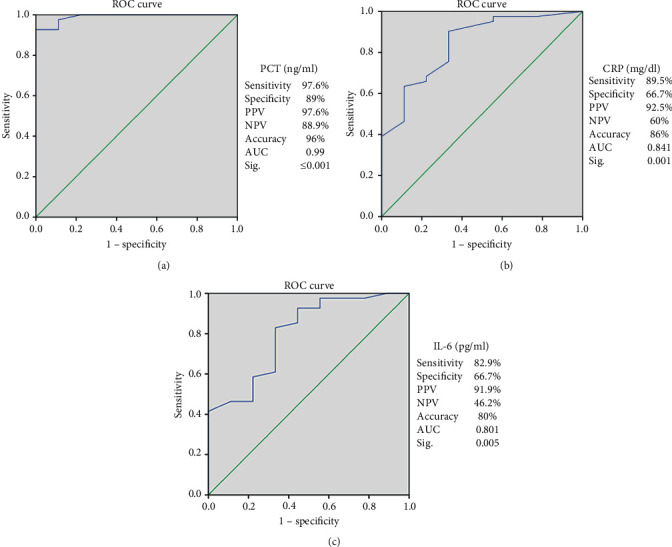
Receiver operating characteristics (ROC) curves for the studied sepsis markers: (a) ROC curve for PCT: AUC: 0.991, *p* value ≤0.001. (b) ROC curve for CRP: AUC: 0.841, *p* value = 0.001 (c) ROC curve for IL-6: AUC: 0.801, *p* value = 0.005. PCT: procalcitonin, CRP: C-reactive protein, IL-6: interleukin 6, PPV: positive predictive value, NPV: negative predictive value, and AUC: area under the curve. Diagonal segments are produced by ties.

Receiver Operating Characteristics (ROC) curves for the studied sepsis markers are shown in Figure 2, which revealed that serum PCT levels with cutoff value ≥0.5 ng/ml showed sensitivity 97.6%, specificity 89%, PPV 97.6%, NPV 88.9%, and a highly significant accuracy 96% (*p* value ≤0.001). Cutoff value of CRP ≥10 mg/dl showed sensitivity 89.5%, specificity 66.7%, PPV 92.5%, NPV 60%, and a significant accuracy 86% (*p* value = 0.001). Cutoff value for IL-6 25 pg/ml showed sensitivity 82.9%, specificity 66.7%, PPV 91.9%, NPV 46.2%, and a significant accuracy 80% (*p* value = 0.005). Also, the levels of IL-6 and CRP in cases at an extreme range of PCT are shown in Table S1 in the supplementary section.

**Figure 3:**
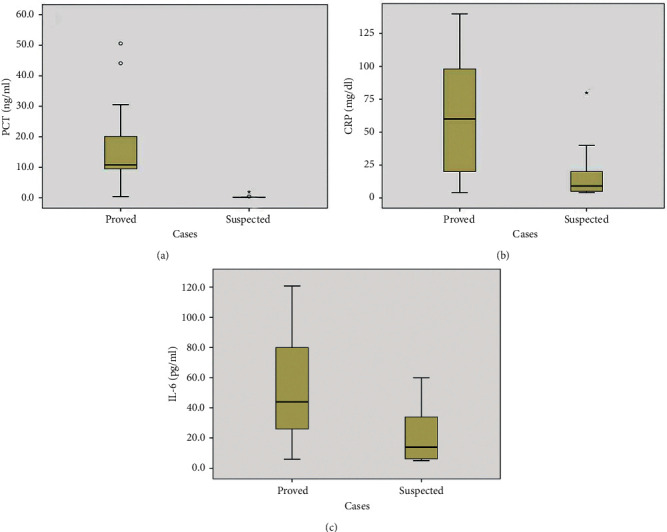
Serum levels of PCT (a), CRP (b), and IL-6 (c) in both proved (*n* = 41) and suspected cases (*n* = 9). Data are presented as box plots with median lines, 25-, 75- quartiles, and outliers, demonstrating how the data were spread across the range.

When analyzing the differences in sepsis markers of the proved and suspected cases of neonatal sepsis by using Mann–Whitney test, the median serum level of PCT was 10.8 ng/ml and ranged 0.4–50.6 ng/ml in the proved cases compared with a median of 0.1 ng/ml and a range of 0.1–1.9 ng/ml in the suspected cases which was highly significant (*p* ≤ 0.001)). For the CRP, the median serum level was 60 mg/dl in the proved cases with a range of 4–140 mg/dl versus a median serum level of 9 mg/dl and a range of 4–80 mg/dl, which was significant (*p*=0.001). Lastly, IL-6 also showed a significant difference between proved and suspected cases (*p*=0.004) with a median serum level of 44 pg/ml and ranged from 6 to 120.8 pg/ml in the proved cases versus a median of 14 pg/ml and a range of 5–60 pg/ml in the suspected cases (Figure 3).

No significant differences were detected for PCT, CRP, nor IL-6 in both gender groups (*p*=0.492, 0.774, and 0.652), respectively (Table 6).

## 4. Discussion

Rapid diagnosis of neonatal sepsis is considered a very important issue to prevent its serious outcome. Many sepsis markers are immerging to help the early diagnosis. Procalcitonin and varieties of proinflammatory cytokines play a role in bacterial sepsis [1]. So, in this study, we were interested in evaluating the CRP, PCT, and IL-6 as sepsis markers for early diagnosis of sepsis in neonates.

This study was done on 50 neonates with clinically suspected sepsis; 33 males and 17 females, their ages ranged from 1 to 16 days with a mean age of 6 ± 2.4, 54% were of low birth weight, 18% had a history of premature rupture of membranes (PROM), and 14% were mechanically ventilated.

In this study, neonatal sepsis was more prevalent in males. This could be due to the X-linked immunoregulatory gene factor which contributes to the susceptibility to infections in them [10]. We found neonatal sepsis more frequently in neonates delivered by spontaneous vaginal delivery, which might originate from the birth canal flora. Premature rupture of membrane (PROM) and preterm labor were also important risk factors for neonatal sepsis. Preterm neonates were more prone to infection. This might be due to an inherent defective mechanism. Moreover, neonates of birth weight less than 2.5 kg showed increased susceptibility to sepsis.

In this study, blood culture revealed pathogenic growth in 31 cases; The most common were *Klebsiella* (61.3%), *E. coli* (9.7%), followed by CONS (9.7%), GBS with 6.5%. *Staphylococcus* aureus, *Citrobacter*, and *Acinetobacter* were less common with 3.2% for each, and finally *Candida albicans* with 3.2%. These results were following the results of other studies carried out in Egypt by Rashwan and coworkers [3], who found that *Klebsiella* and *Staphylococcus aureus* were the most common Gram-negative and Gram-positive isolates, respectively. Also, El-Behedy et al. [11] in Egypt reported that *Klebsiella* had the greatest incidence (40%). Al-Zahrani et al. [5] in Saudi Arabia found that Gram-negative organisms (*Klebsiella* and *E. coli*) were the most common, followed by GBS.

Organisms causing sepsis vary in developing countries from the developed ones. In developing countries, Gram-negative bacilli were the most common agents. This may be due to maternal intrapartum antimicrobial prophylaxis in these countries, which leads to decreased GBS infection rates, and the neonates still can catch these Gram-negative organisms from the vaginal and fecal flora of the mother. However, other studies in developed countries carried out by Ozkan et al. [12] and Simonsen et al. [13] reported that CONS and GBS were the most common agents and added that they cause low mortality rate than Gram-negative bacilli.

Two of our positive blood cultures were found to be, negative for the PCR results of bacterial 16S rDNA, which might reflect low-level bacteremia with a low detection limit of the PCR technique. This was in keeping with the observation of Reier-Nilsen et al. [14], who found the detection limit of PCR to be 10^3^–10^4^ CFU/ml, which was not allowing detection of low-level bacteremia. Also, we found 10 positive PCR samples, despite negative blood culture. This could be due to an inadequate amount of blood samples that allow the optimal detection of bacteria. However, collecting larger samples is not an option in neonates. So, bacteremia of low level in neonates would be difficult to be identified by any procedure based on the detection of bacterial growth or DNA, which raises the need for neonatal sepsis markers.

These results were in keeping with those of Al-Zahran and coworkers [5], who reported two samples positive in blood culture and negative for PCR, and seven samples with positive PCR that were negative in blood culture. Also, Reier-Nilsen et al. [14] reported a patient with a positive blood culture who had a negative PCR result; and six neonates with positive PCR and negative blood cultures.

Because the use of blood culture as a gold standard for diagnosis of sepsis has been an issue of debate, the studied neonates were divided into two groups: the proved sepsis group involved neonates with positive blood culture and/or positive PCR results (*n* = 41), and the suspected sepsis group contained neonates suspected of having sepsis with negative both blood culture and PCR (*n* = 9).

The occurrence of culture-proven sepsis was in 31 out of 41 cases (75.6%). Those missed cause lack of confidence in negative blood culture results, which may be due to the inoculation of a small amount of blood together with the fact that about 60%–70% of neonates had low bacteremia levels. So, for better results, we need 6–10 ml of blood, which is not feasible in neonates. Therefore, other sepsis markers must be included.

PCR-proven sepsis was detected in 39/41 cases (95.1%), and nearly similar results were reported by Reier-Nilsen et al. [14]. Although PCR shows higher sensitivity and may represent an optimal method for sepsis diagnosis, actually it lacks reporting the antibiotic sensitivity profile for the organisms. Also, PCR gives positive results even with antibiotic therapy as it does not depend on the viability of bacteria. So, we are still in need for other markers.

Receiver operating characteristics (ROC) curves for our studied sepsis markers revealed that serum PCT levels with cutoff value ≥0.5 ng/ml showed the highest sensitivity, specificity, PPV, NPV, and accuracy of 97.6%, 89%, 97%, 88.9%, and 96% than other studied markers. Our results are keeping with that reported by Adib and his colleagues [1], who found that sensitivity, specificity, and positive and negative predictive values of PCT level for neonatal sepsis were 75%, 80%, 80%, and 75%, respectively. Several studies also found the PCT a promising marker for neonatal sepsis diagnosis in which the PCT sensitivity ranged from 83% to 100%, and the specificity ranged from 70% to 100% [2, 15]. By contrast, Stocker and his colleagues [16] reported that PCT level could also be increased in noninfected neonates with either hemorrhage, pneumothorax, or perinatal asphyxia, which might negatively affect the PCT specificity but added that this rise of PCT level was smaller than that against infection.

PCT seems to be a promising sepsis marker in both adults and neonates as it has many advantages. It can discriminate systemic response due to bacterial or fungal causes from others as viral infection, it starts to rise four hours after exposure to bacteria with obtaining its maximum level after six to eight hours and its serum levels are associated with the severity of the infection and decreases rapidly after antibiotic treatment. Finally, PCT can discriminate between true infections and contaminated blood cultures. So PCT is not only useful in the rapid diagnosis but also monitoring treatment response [5]. But it is an expensive test, which was considered a limitation to be used as a routine test in the diagnosis, especially in developing countries.

In the current study, there was a remarkable elevation in the PCT level in the proved group than the suspected neonatal sepsis group (*p* ≤ 0.001). These results were in keeping with the results of Zahedpasha et al. [17], who added a dramatic decrease in the PCT levels after antibiotic treatment. Also, Park et al. [18] intensified our results. Also, among 9 of our suspected neonates, only one had a PCT level above the cutoff value. This could be explained by the increase of PCT physiologically in healthy neonates with peak values at 24–48 hours postnatal [19].

In this study, with a cutoff value of CRP ≥10 mg/dl by the ROC curve, the CRP had a sensitivity (89.5%), a specificity (66.7%), a PPV (92.5%), a NPV (60%), and a significant accuracy 86% (*p* value = 0.001). Moreover, we revealed that CRP levels were elevated in a significant manner in the proved group than the suspected cases (*p*=0.001). Other studies declared that the test sensitivity ranged from 70% to 93%, specificity 41%–98%, PPV 6%–83%, and NPV 97%–99% [2, 3]. Although seeming to have average sensitivity and specificity, but as reported by Çetinkaya and coworkers [20], the increase in CRP levels is slow during the first 24–48 hours of infection, which negatively affects its sensitivity. Also, the rise of the CRP level in noninfected cases badly affects its specificity. So, we suggest, for obtaining the best results, to be combined with other markers and not to be used alone as a sepsis marker.

Hasan and his colleagues [21] added that the advantages of PCT over CRP are that its level increases mainly in bacterial infection, and its normal level is rapidly restored after antibiotic therapy. So, PCT is superior to CRP in the early diagnosis of neonatal sepsis, detecting sepsis severity and evaluating the antibiotic treatment response.

Moreover, depending on the ROC curve analysis, the best cutoff value for IL-6 was 25 pg/ml, which showed a sensitivity of 82.9%, a specificity of 66.7%, a PPV 91.9%, a NPV 46.2%, and a significant accuracy 80%. IL-6 levels were found to be significantly elevated in the proved sepsis group than suspected neonatal sepsis cases (*p*=0.004). Similarly, Noor and coworkers [22] reported IL-6 to have a sensitivity (76.9%), specificity (73.7%), PPV (80%), and NPV (70%), and Al-Zahrani and coworkers [5] reported that IL-6 to have a sensitivity (63.6%) and specificity (69%) and added that it does not cross the placenta and any rise during first few hours postpartum could predict sepsis.

Also, there were no significant differences for PCT, CRP, or IL-6 in both gender groups. So, they are not affected by gender and can be used regardless of sex.

According to this study findings, PCT, CRP, and IL-6 were shown to be highly significantly elevated in the confirmed sepsis cases when compared with the suspected cases, which proved not to be infected by both blood culture and PCR (*p* ≤ 0.001, *p*=0.001, *p*=0.004 respectively). This was following the results declared by Abdollahi et al. [23], who reported that patients with evidence of sepsis had higher levels of PCT, CRP, and IL-6 compared with those with uncertain sepsis. Also, when the levels of our sepsis markers were compared in both male and female groups, no significant differences were detected for PCT, CRP, nor IL-6 (*p*=0.492, 0.774 and 0.652), respectively.

Thanks to its better sensitivity and specificity, we suggest PCT as a good diagnostic marker for neonatal sepsis, helping in the rapid and early diagnosis to initiate early antibiotic therapy and decrease the number of patients treated unnecessarily. This is in agreement with Al-Zahrani and coworkers [5], who suggested that PCT is more accurate than IL-6 and CRP in neonatal sepsis diagnosis. Ruan et al. [6] found the PCT more sensitive than CRP but stated that the use of the two tests in combination would result in better sensitivity and would be more helpful in sepsis diagnosis. Rashwan and coworkers [3] reported that it would be better to use a combination of PCT and IL-6 than a single marker.

We indeed did not ignore the main limitations in this study, which were the time point correlations among our sepsis markers and lacking the follow-up to allow a prognostic estimation and face the clinical challenges. Previous studies showed a correlation between the PCT and the CRP levels in the proved sepsis group after 12–24 h of admission in contrast to IL-6, which had a very short half-life and became undetectable 24 hours after the onset of infection [23].

In our opinion, although PCT was the best diagnostic marker, due to being expensive, we advise restricting using it in neonatal sepsis cases with clinical symptoms of sepsis with negative CRP to prove sepsis.

## 5. Conclusion

Procalcitonin is a better sepsis marker than CRP and IL-6. Including PCT in neonatal sepsis diagnosis will be useful in starting antibiotics as early as possible and limiting the unnecessary use of antibiotics, which affects the development of resistant bacteria. This would be useful in reducing the number of intensive care neonatal admission, shortening hospital stay, and increasing the availability of hospital beds, which are very important issues, especially in developing countries with limited facilities.

## Figures and Tables

**Table 1:** Primer set used for PCR.

Primer sequence (5′-3′)	Product size (bp)	Reference
F: TGAAGAGTTTGATCATGGCTCAG	500	[5]
R: TACCGCGGCTGCTGGCA		

**Table 2:** Demographic and laboratory data of the studied group.

Data	Studied group
Age (days)	
Mean ± SD	6 ± 2.4
Range	(1–16)
Sex	
Male/female ratio	33/17
Gestational age, *n* (%)	
Preterm	19 (38%)
Full-term	31 (62%)
Mode of delivery, *n* (%)	
NVD	28 (56%)
CS	23 (46%)
PROM, *n* (%)	
Yes	9 (18%)
No	41 (82%)
Low birth weight, *n* (%)	
Yes	27 (54%)
No	23 (46%)
Mechanical ventilation, *n* (%)	
Yes	7 (14%)
No	43 (86%)
TLC (1000/mm^3^)	
Mean ± SD	22 ± 8.7
Range	(3.5–35)
I/T ratio	
Mean ± SD	0.41 ± 0.17
Range	(0.1–0.8)

NVD: normal vaginal delivery, CS: cesarean section, PROM: premature rupture of membranes, TLC: total leukocytic count, and I/T ratio: immature/T cell ratio.

**Table 3:** Comparison of blood culture and PCR results of the studied neonates.

PCR	Blood culture		
	+ve	−ve	Total
+ve	29	10	39
−ve	2	9	11

Total	31	19	50

**Table 4:** Comparison of blood culture results with proved sepsis.

Blood culture	Proved sepsis		
	+ve	−ve	Total
+ve	31	0	31
−ve	10	9	19

Total	41	9	50

**Table 5:** Comparison of PCR results with proved sepsis.

PCR	Proved sepsis		
	+ve	−ve	Total
+ve	39	0	39
−ve	2	9	11

Total	41	9	50

**Table 6:** Differences in sepsis markers according to gender.

	Male group (*n* = 33)	Female group (*n* = 17)	Sig.
PCT (ng/ml)			
Median	10.3	10.6	0.492^∗^
Range	0.1–50.6	0.2–26.4	
CRP (mg/dl)			
Median	60	44	0.774^∗^
Range	4–140	5–110	
IL-6 (pg/ml)			
Median	36	36.2	0.652^∗^
Range	5–120	0.6–120.8	

^∗^Mann–Whitney test: *p* > 0.05 is not significant.

## Data Availability

All the data used to support the study are included within the article and no other data have been specified to be uploaded in any database.

## Supplementary Materials

Supplementary Materials Table (S1): the levels of IL-6 and CRP in cases at an extreme range of PCT.
