# Supplementary material for: Evaluation of Procalcitonin, C-Reactive Protein, and Interleukin-6 as Early Markers for Diagnosis of Neonatal Sepsis
Source: Int J Microbiol. 2020 Oct 1;2020:8889086. doi: 10.1155/2020/8889086 (PMC7547329; doi:10.1155/2020/8889086)
Supplement: Supplementary Materials — Table (S1): the levels of IL-6 and CRP in cases at an extreme range of PCT. [file 8889086.f1.doc]

Table (S1): The levels of IL-6 and CRP in cases at an extreme range of PCT.

| **Cases** | **PCT** | **CRP** | **IL-6** |
| --- | --- | --- | --- |
| **1** | 0.1 | 9 | 60 |
| **2** | 50.6 | 140 | 22 |
| **3** | 44.1 | 20 | 70 |
| **4** | 0.1 | 4 | 6 |
| **5** | 0.2 | 5 | 10 |
| **6** | 0.1 | 6 | 14 |
| **7** | 0.1 | 9 | 24 |

Concerning the results in that table, in the first case, PCT was low despite high CRP and IL-6 levels, which could be explained by the presence of any other inflammatory conditions. This was a suspected case with both PCR and culture negative.

In case two, both PCT and CRP are increased, but IL-6 was at a normal level. This can be explained by late sampling as IL-6 increases rapidly and decreases rapidly.

In case three, although PCT, IL-6 were high, CRP was normal. This can be explained by early sampling as CRP increases late.

In cases from 4 to 7, there was a low level of PCT, CRP, and IL-6 in suspected cases of sepsis.
